# Strong Epistatic Selection on the RNA Secondary Structure of HIV

**DOI:** 10.1371/journal.ppat.1004363

**Published:** 2014-09-11

**Authors:** Raquel Assis

**Affiliations:** Department of Biology, Huck Institutes of the Life Sciences, Center for Medical Genomics, Pennsylvania State University, University Park, Pennsylvania, United States of America; University of Michigan, United States of America

## Abstract

A key question in evolutionary genomics is how populations navigate the adaptive landscape in the presence of epistasis, or interactions among loci. This problem can be directly addressed by studying the evolution of RNA secondary structures, for which there is constraint to maintain pairing between Watson-Crick (WC) sites. Replacement of a nucleotide at one site of a WC pair reduces fitness by disrupting binding, which can be restored via a compensatory replacement at the interacting site. Here, I present the first genome-scale analysis of epistasis on the RNA secondary structure of human immunodeficiency virus type 1 (HIV-1). Comparison of polymorphism frequencies at ancestrally conserved sites reveals that selection against replacements is ∼2.7 times stronger at WC than at non-WC sites, such that nearly 50% of constraint can be attributed to epistasis. However, almost all epistatic constraint is due to selection against conversions of WC pairs to unpaired (UP) nucleotides, whereas conversions to GU wobbles are only slightly deleterious. This disparity is also evident in pairs with second-site compensatory replacements; conversions from UP nucleotides to WC pairs increase median fitness by ∼4.2%, whereas conversions from GU wobbles to WC pairs only increase median fitness by ∼0.3%. Moreover, second-site replacements that convert UP nucleotides to GU wobbles also increase median fitness by ∼4%, indicating that such replacements are nearly as compensatory as those that restore WC pairing. Thus, WC peaks of the HIV-1 epistatic adaptive landscape are connected by high GU ridges, enabling the viral population to rapidly explore distant peaks without traversing deep UP valleys.

## Author Summary

Epistasis is an evolutionary process in which the effect of a nucleotide at one site in the genome is dependent on the presence or absence of particular nucleotides at other sites in the genome. One of the simplest types of epistasis occurs between Watson-Crick (WC) nucleotides in RNA secondary structures, which are under constraint to maintain base-pairing. In this study, I examine the effects of mutations at WC sites in the RNA secondary structure of HIV-1. I show that while epistasis plays a major role in the evolution of the HIV-1 secondary structure, different types of mutations have variable effects on fitness. Therefore, by favoring certain mutational trajectories, HIV-1 can evolve rapidly despite strong epistatic constraint on its RNA secondary structure.

## Introduction

Epistasis is an evolutionary phenomenon whereby the fitness effect of a mutation is conditional on the genetic background in which it arises [1], [2]. One of the simplest forms of epistasis occurs between WC sites of RNA secondary structures. Replacement of a nucleotide at one site of a WC pair is often deleterious because it disrupts binding, decreasing the stability of the RNA secondary structure. However, a second-site replacement in the modified genetic background may be beneficial, or compensatory, if it restores binding by creating a new WC pair [3]. Due to these complex interactions, RNA secondary structures evolve along rugged, or multi-peaked, adaptive landscapes [4], on which certain mutational trajectories may be inaccessible due to highly deleterious intermediate states. Thus, understanding how a population navigates from one peak to another on an epistatic adaptive landscape is a fundamental problem in evolutionary biology.

Selection to maintain WC pairing in RNA secondary structures leaves distinct evolutionary footprints. For one, theoretical work shows that evolutionary rates should be lower at WC than at unpaired sites [5], [6], a pattern that has been observed in noncoding and synonymous regions of many viral RNA secondary structures, including those of influenza A [7], hepatitis C [8], [9], and HIV-1 [10]–[12]. The negative correlation between extent of WC pairing and amino acid variability in HIV-1 suggests that selection to maintain WC pairing may also decrease evolutionary rates at nonsynonymous WC sites [13]. Moreover, because transitions occur more frequently and are thus compensated more rapidly than transversions, transition-to-transversion ratios are elevated at WC sites of RNA secondary structures [14]. Thus, epistatic selection can significantly alter the genomic landscape by modulating the numbers and types of mutations at WC sites of RNA secondary structures. These signatures of epistatic selection have been used both to predict and evaluate RNA secondary structures [14]–[16].

Epistatic interactions between WC sites have been studied in a variety of RNA molecules, including mRNAs [3], [17], rRNAs [18]–[20], tRNAs [21], [22], and RNA viruses [7], [23]–[41]. These analyses have uncovered several key evolutionary principles. First, constraint to maintain WC pairing can result in strong long-term conservation of RNA secondary structures, yet weak conservation at the nucleotide level [18], [24]. A striking example of this phenomenon involves the nearly identical secondary structures of the R regions of HIV-2 and simian immunodeficiency virus in mandrills, which have highly conserved WC pairing interactions despite a sequence homology of only 40% [24]. Second, introduction of a mutation at a WC site typically results in impaired function, decreased thermodynamic stability, and lower fitness of a RNA secondary structure [3], [5], [7], [17], [20]–[23], [25]–[41]. Third, compensatory replacements at WC sites often fully restore the function, thermodynamic stability, and fitness of a RNA secondary structure [3], [17], [21]–[23], [27], [28], [30], [32]–[35], [38]–[41]. Fourth, second-site compensatory replacements may be preferred over back mutations [23], an intriguing finding that is also supported by studies of compensatory evolution in other interaction schemes [42]–[44]. Finally, compensatory evolution often proceeds through GU wobble intermediates [3], [19], [22], [23], which are nearly as thermodynamically stable as WC pairs and are ubiquitous in RNAs from organisms in all three domains of life [23], [45]. In some cases, GU wobbles may even confer higher fitness than WC pairs, resulting in their long-term retention [18], [19].

While the dynamics of WC pairing have been extensively studied in HIV-1 [24]–[41], previous analyses primarily focused on secondary structures located in the 5′LTR, which regulates the transcription of viral genes. Little is known about the evolution of secondary structures across the HIV-1 genome. Recently, the RNA secondary structure of the entire HIV-1 subtype B NL4-3 genome was experimentally derived with high confidence via high-throughput selective 2′-hydroxyl acylation analyzed by primer extension (SHAPE) reactivity [46]. The availability of this structure provides a novel opportunity to study the evolution of WC pairing in HIV-1 on a genome-wide scale.

HIV-1 is an ideal model system in which to study epistasis at WC sites for a number of reasons. First, there is an abundance of publicly available sequence data for HIV-1. Second, HIV-1 has one of the highest observed spontaneous mutation rates and a relatively small genome and, thus, the waiting time for new mutations is short [47]. Third, experimental analyses of HIV-1 have demonstrated the importance of its RNA secondary structure at all stages of the viral life cycle, including reverse transcription [33], [35], frameshifting [36]–[38], mRNA splicing [39], [41], and viral packaging and transport [26], [31], [33]. Mutations that disrupt WC pairing in important domains often have severe phenotypic consequences [25]–[41], and site-directed mutagenesis studies have shown that compensatory mutations that re-establish WC pairing can restore wild type functions [27], [28], [30], [32]–[35], [38]–[41]. Finally, HIV-1 is a virus of great clinical significance, and knowledge about its evolutionary dynamics at the structural level may inform public health studies.

## Results and Discussion

To investigate epistatic interactions between WC sites of HIV-1, I utilized the subtype B NL4-3 genomic sequence and RNA secondary structure [38] as a reference, 197,863 subtype B sequences (1,867 genomic) for intra-population comparison, and 66 subtype D (closest relative to subtype B) genomic sequences as an outgroup. Pairing probabilities associated with the RNA secondary structure [38] were not used because they were computed via phylogenetic analyses of covariation between sites. I restricted my analysis to sites at which reference nucleotides are ancestral, *i.e.*, conserved in all 66 outgroup sequences, both enabling polarization of mutations in the subtype B population and ensuring that reference nucleotides have been under long-term selective constraint and are therefore likely important to the RNA secondary structure. Additionally, I only considered noncoding and synonymous sites so as not to confound selection on pairing with selection on amino acid composition, though nonsynonymous sites were also analyzed separately (see Materials and Methods). Using these criteria, I identified 562 WC sites (281 pairs) and 2,868 non-WC (nWC) sites in the secondary structure of the HIV-1 reference genome.

Stems utilized in this analysis have a median length of 4 bp and are distributed across the HIV-1 genome (Table 1). Most stems are located in *Pol*, which is the gene responsible for transcribing viral RNA into double-stranded DNA, and which also contains the greatest number of ancestral WC sites. Surprisingly, while most studies of WC pairing interactions in HIV-1 have focused on the 5′LTR, this region contains the fewest number of stems, indicating that much information may be gained from studying the evolution of WC pairing across the entire HIV-1 genome.

**Table 1:** Number of stems, median stem size, and number of ancestral WC sites in different regions of the HIV-1 genome.

	Number of stems	Median stem size (bp)	Number of ancestral WC sites
**5′LTR**	13	4	101
**Gag**	78	4	66
**Pol**	133	4	119
**Vif**	24	4.5	25
**Vpr**	19	4	13
**Tat**	24	3	16
**Rev**	24	3	21
**Vpu**	15	5	17
**Env**	125	4	87
**Nef**	37	4	24
**3′LTR**	34	4	104

Note that many genomic regions of HIV-1 overlap.

Comparisons of subtype B sequences to the reference sequence yielded 1,105 polymorphisms at WC sites and 7,723 polymorphisms at nWC sites. These counts indicate that there are, on average, 1.97 polymorphisms per WC site and 2.69 polymorphisms per nWC site. Thus, because there are three possible replacement nucleotides per site, WC sites are ∼66% saturated and nWC sites are ∼90% saturated, and this difference is highly significant (*p*<2.20×10^−16^, Binomial test; see Materials and Methods for details). Additionally, of the 1,105 WC polymorphisms, only 306 occur as single-site replacements; the remaining 799 polymorphisms correspond to 669 double-site replacements (see Materials and Methods). Together, the lower mutational saturation at WC sites and tendency for polymorphisms at interacting WC sites to co-segregate highlight the importance of epistasis in the evolution of the HIV-1 secondary structure.

**Figure 1:**
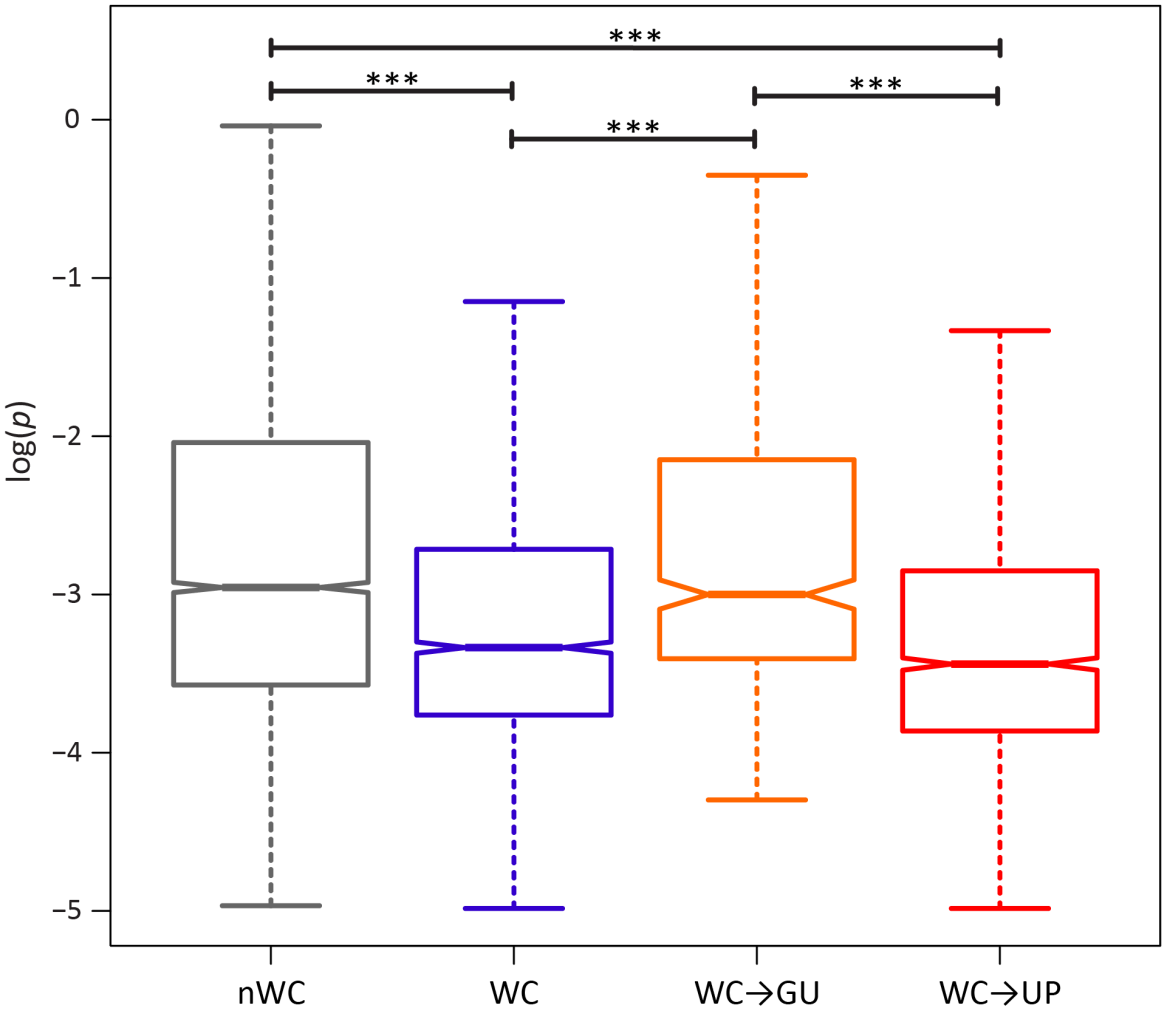
Intra-population frequencies of nWC and single-site WC replacement polymorphisms in the HIV-1 genome. Frequencies are normalized to enable comparisons among classes (see Materials and Methods for details) and plotted on a log_10_-scale. Asterisks indicate *p*<0.05 (*), *p*<0.01 (**), and *p*<0.001 (***).

If conservation of WC interactions in the HIV-1 secondary structure is important, destruction of WC pairing should result in a significant fitness loss. Comparison of the intra-population frequencies of single-site WC polymorphisms to those of nWC polymorphisms revealed that this is indeed the case (Figure 1). WC polymorphisms segregate at frequencies that are ∼82% lower than those of nWC polymorphisms, and this difference is highly significant (*p* = 1.07×10^−8^, Mann-Whitney *U* test), indicating that there is strong selection against destruction of WC pairing in the HIV-1 secondary structure. To estimate the strength of this constraint, I computed selection coefficients against WC and nWC polymorphisms by *s* = *μ*/*p_med_* [48], where *μ* is mutation rate, which has been estimated as 3.0×10^−5^ replacements/site/replication cycle [47], and *p_med_* is the median frequency of the segregating polymorphism (Table 2). Differences between selection coefficients against WC and nWC polymorphisms indicate that ∼63% of constraint at WC sites, and ∼46% of genome-wide constraint, is due to epistatic interactions between nucleotides in the RNA secondary structure of HIV-1 (see Materials and Methods for details).

**Table 2:** Median frequencies, selection coefficients, and relative fitnesses of nWC and single-site WC replacement polymorphisms in the HIV-1 genome.

	*p_med_*	*s*	*w_rel_*
**nWC sites**	2.37×10^−3^	1.26×10^−2^	0.987
**WC sites**	8.76×10^−4^	3.43×10^−2^	0.966
**WC→GU**	2.04×10^−3^	1.47×10^−2^	0.985
**WC→UP**	6.47×10^−4^	4.64×10^−2^	0.954

A single replacement at a WC site can either result in two unpaired nucleotides (WC→UP) or in a GU pair (WC→GU). Because previous studies have demonstrated that WC→GU replacements are typically slightly deleterious, and can sometimes even be beneficial, selection against WC→GU replacements should be weaker than selection against WC→UP replacements [3], [19], [22], [23], [45]. Consistent with this expectation, WC→GU polymorphisms segregate at significantly higher frequencies than WC→UP polymorphisms (*p* = 3.75×10^−6^, Mann-Whitney *U* test; Figure 1), such that selection against WC→UP replacements is approximately three times stronger than selection against WC→GU replacements (Table 2). Moreover, frequencies of WC→GU polymorphisms are comparable to those of nWC polymorphisms (*p* = 0.86, Mann-Whitney *U* test), and selection against WC→GU replacements is marginally stronger than selection against replacements at nWC sites (see Table 2), indicating that WC→GU replacements are slightly deleterious in the HIV-1 secondary structure.

**Figure 2:**
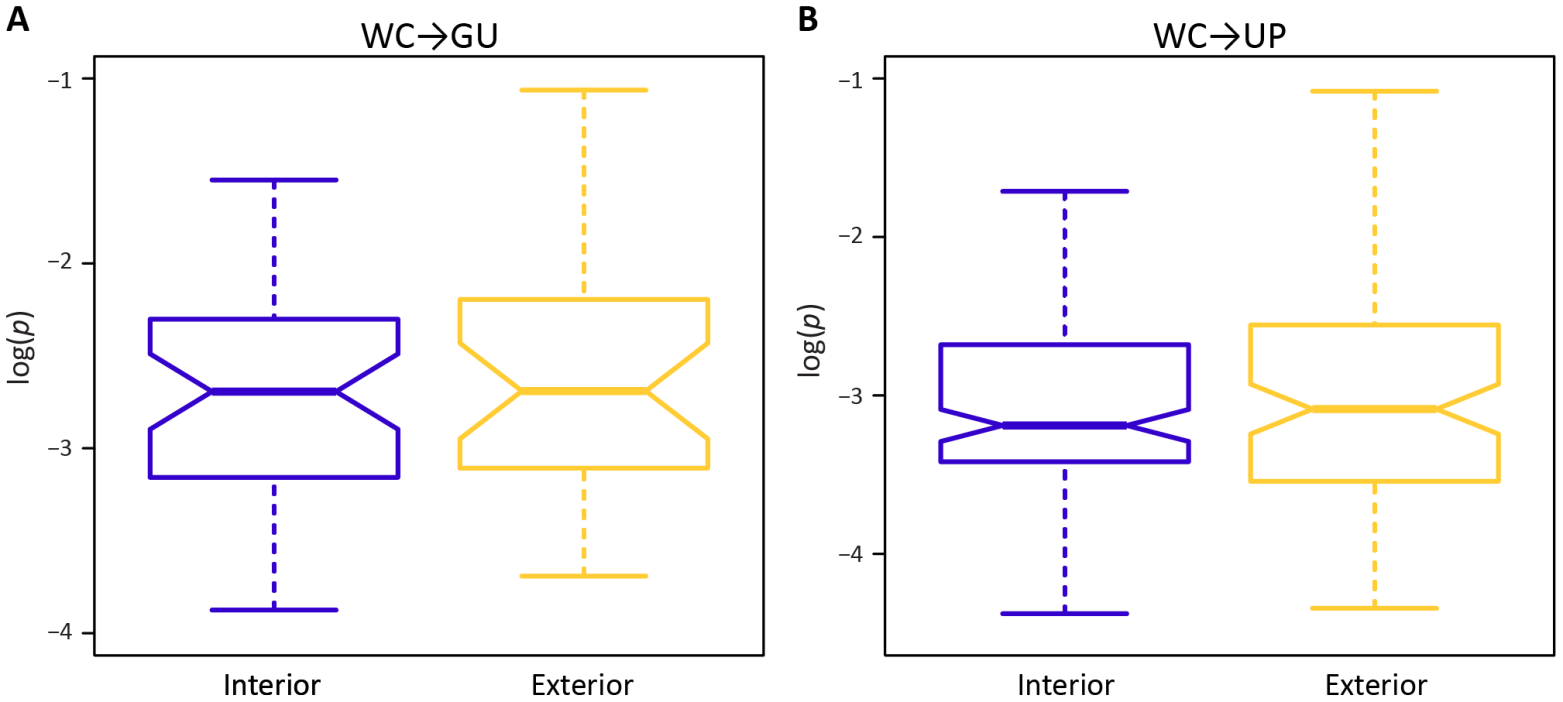
Intra-population frequencies of single-site WC replacement polymorphisms at interior and exterior stem positions. *A*) Frequencies of WC→GU polymorphisms. *B*) Frequencies of WC→UP polymorphisms. Frequencies are normalized to enable comparisons among classes (see Materials and Methods for details) and plotted on a log_10_-scale. Asterisks indicate *p*<0.05 (*), *p*<0.01 (**), and *p*<0.001 (***).

**Figure 3:**
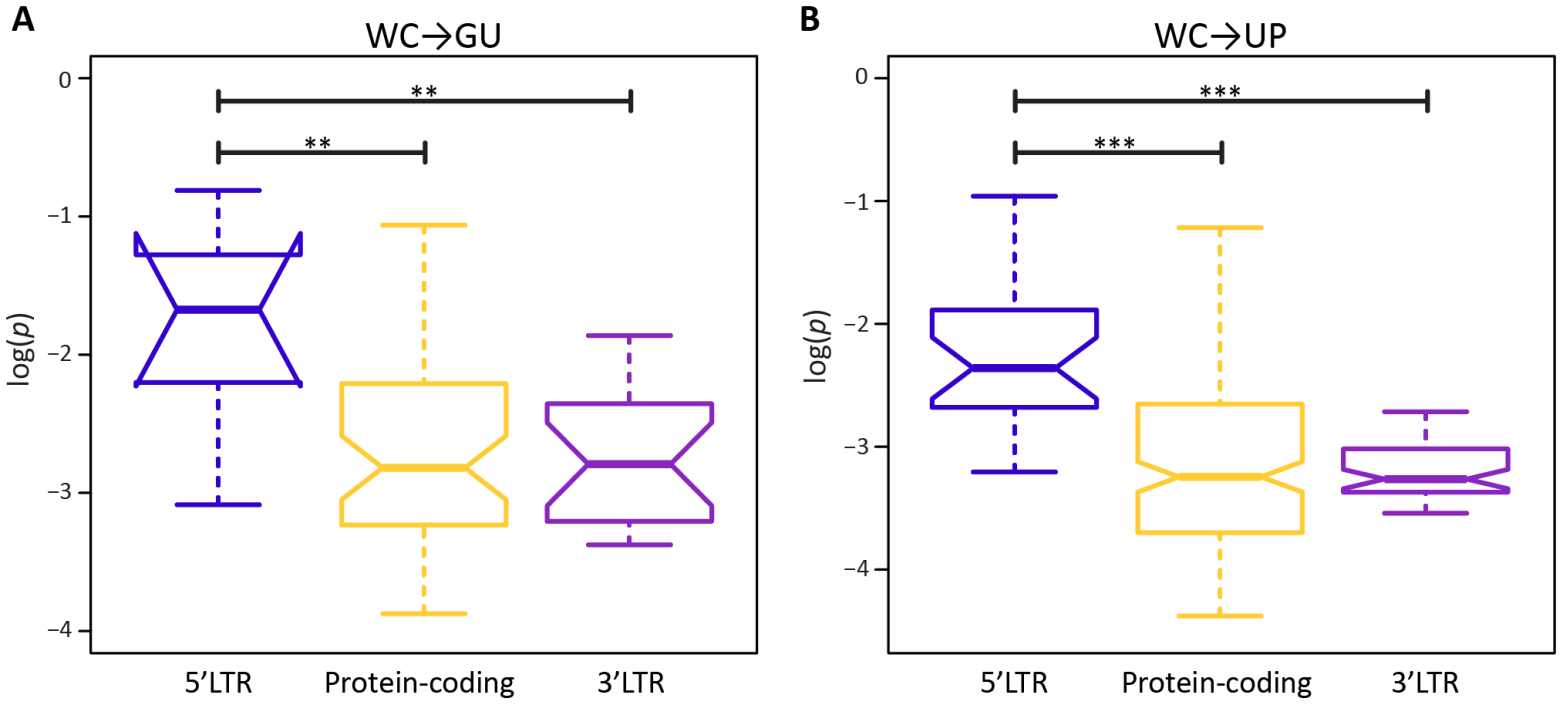
Intra-population frequencies of single-site WC replacement polymorphisms in 5′LTR, protein-coding, and 3′LTR regions of the HIV-1 genome. *A*) Frequencies of WC→GU polymorphisms. *B*) Frequencies of WC→UP polymorphisms. Frequencies are normalized to enable comparisons among classes (see Materials and Methods for details) and plotted on a log_10_-scale. Asterisks indicate *p*<0.05 (*), *p*<0.01 (**), and *p*<0.001 (***).

A potential factor in the effect of a mutation at a WC site is location. In particular, location-specific effects of replacements may be due to position within a stem or within the HIV-1 genome. Surprisingly, frequencies of both WC→GU and WC→UP polymorphisms are similar for interior and exterior stem sites (*p* = 0.65, *p* = 0.97, Mann-Whitney *U* tests; Figure 2). However, frequencies of both replacement classes differ among sites located in 5′LTR, protein-coding, and 3′LTR regions of the HIV-1 genome (Figure 3). In particular, WC→GU and WC→UP polymorphisms segregate at significantly higher frequencies in 5′LTR than in either protein-coding (*p* = 8.01×10^−3^, *p* = 5.49×10^−7^, Mann-Whitney *U* tests) or 3′LTR (*p* = 5.20×10^−3^, *p* = 7.05×10^−8^, Mann-Whitney *U* tests) regions, which contain similar distributions of polymorphism frequencies (*p* = 0.94, *p* = 0.33, Mann-Whitney *U* tests). Thus, WC sites in the 5′LTR are less constrained than those in any other genomic region, underlining the importance of regulatory changes in the evolution of HIV-1.

**Figure 4:**
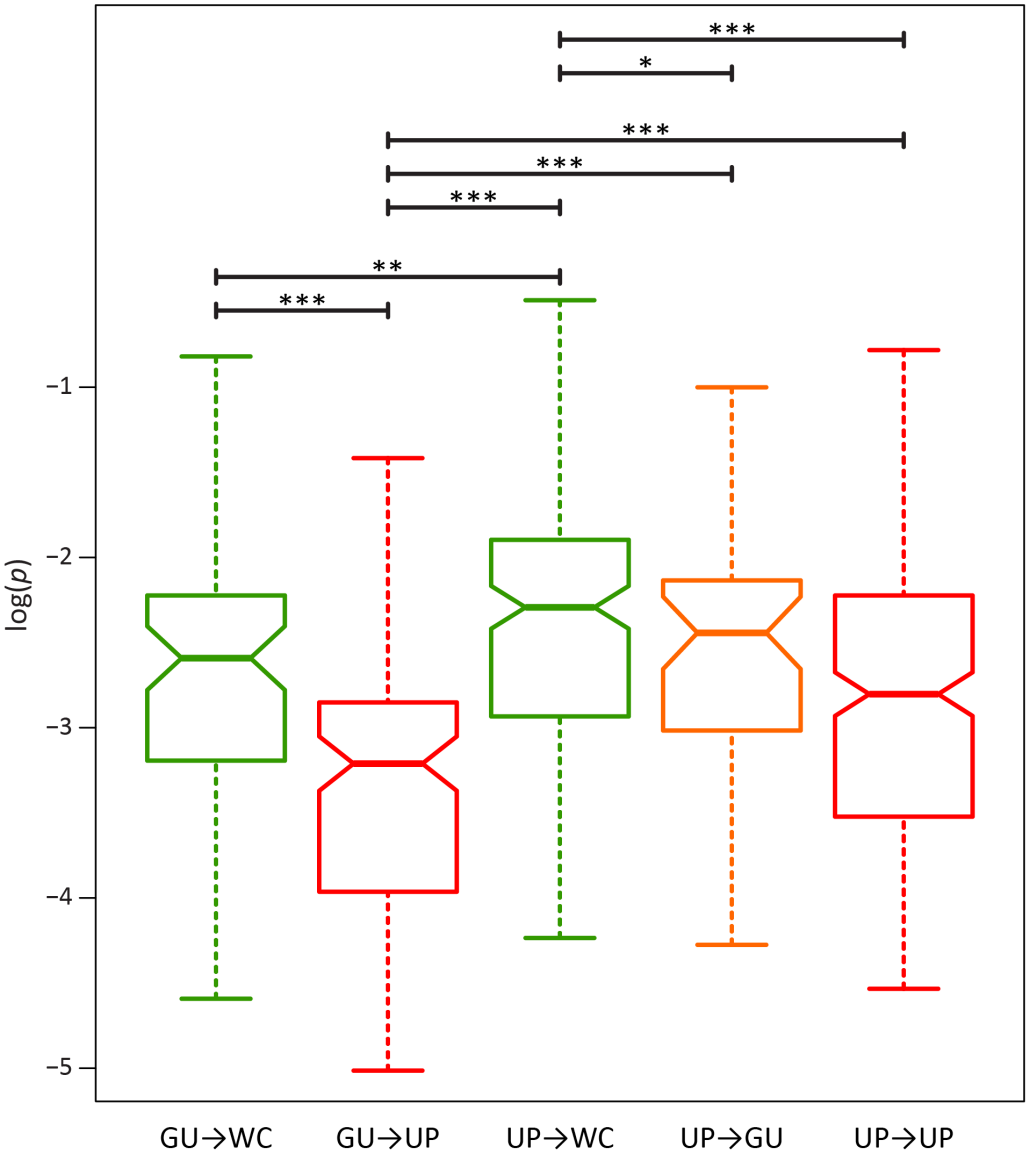
Intra-population frequencies of second-site WC replacement polymorphisms in the HIV-1 genome. Frequencies are normalized to enable comparisons among classes (see Materials and Methods for details) and plotted on a log_10_-scale. Asterisks indicate *p*<0.05 (*), *p*<0.01 (**), and *p*<0.001 (***).

To further elucidate the nature of epistatic interactions at WC sites of the HIV-1 secondary structure, I investigated the fitness effects of second-site WC replacements. A second-site replacement after an initial WC→GU replacement can result in a WC pair (GU→WC) or unpaired nucleotides (GU→UP), while a second-site replacement after an initial WC→UP replacement can result in a WC pair (UP→WC), a GU wobble (UP→GU), or unpaired nucleotides (UP→UP). Consistent with the prediction that restoration of WC pairing is compensatory, GU→WC polymorphisms segregate at significantly higher frequencies than GU→UP polymorphisms (*p* = 2.44×10^−5^, Mann-Whitney *U* test), and UP→WC polymorphisms segregate at significantly higher frequencies than either UP→GU (*p* = 0.04, Mann-Whitney *U* test) or UP→UP (*p* = 8.21×10^−7^, Mann-Whitney *U* test) polymorphisms (Figure 4). However, frequencies of UP→WC polymorphisms are also significantly greater than those of GU→WC polymorphisms (*p* = 4.07×10^−3^, Mann-Whitney *U* test; Figure 4). Moreover, while UP→WC replacements increase median fitness by ∼4.2% relative to initial WC→UP replacements, GU→WC replacements only increase median fitness by ∼0.3% relative to initial WC→GU replacements (see Materials and Methods for details). This difference indicates that conversion of a GU wobble back to a WC pair results in a small fitness gain that is comparable to the small fitness loss from an initial WC→GU replacement.

Because GU wobbles confer higher fitness than UP nucleotides in the HIV-1 secondary structure, the difference between frequencies of second-site GU→WC and GU→UP polymorphisms may be due not only to the compensatory nature of second-site GU→WC replacements, but also to fitness losses from second-site GU→UP replacements. To test this hypothesis, I compared the frequencies of second-site GU→UP and UP→UP polymorphisms, since the latter second-site replacement should not result in a fitness loss relative to the initial WC→UP replacement. Indeed, GU→UP polymorphisms segregate at significantly lower frequencies than UP→UP polymorphisms (*p* = 2.42×10^−5^, Mann-Whitney *U* test), such that selection against GU→UP replacements is ∼2.6 times stronger than selection against UP→UP replacements (see Table 3). Moreover, second-site GU→UP replacements result in a median fitness loss of ∼3.5% relative to initial WC→GU replacements (see Materials and Methods for details), illustrating the highly deleterious effect of losing all complementarity at ancestral WC sites in the HIV-1 secondary structure.

**Table 3:** Median frequencies, selection coefficients, and relative fitnesses of second-site WC replacement polymorphisms in the HIV-1 genome.

	*p_med_*	*s*	*w_rel_*
**GU→WC**	2.56×10^−3^	1.17×10^−2^	0.988
**GU→UP**	6.15×10^−4^	4.88×10^−2^	0.951
**UP→WC**	5.09×10^−3^	5.90×10^−3^	0.994
**UP→GU**	3.60×10^−3^	8.32×10^−3^	0.992
**UP→UP**	1.57×10^−3^	1.91×10^−2^	0.981

While restoration of WC pairing is thought to be the only mechanism for compensatory evolution, the findings from this study prompt a key question: Are second-site UP→GU replacements also compensatory in the HIV-1 secondary structure? Examination of frequencies of UP→GU polymorphisms shows that they are intermediate to those of UP→UP and UP→WC polymorphisms (Figure 4). Thus, second-site UP→GU replacements are indeed compensatory, though not to the degree of UP→WC replacements. In particular, UP→GU replacements result in a ∼4% fitness gain relative to initial WC→UP replacements, in contrast to the ∼4.2% fitness gain conferred by UP→WC replacements (see Materials and Methods for details). Hence, as expected given the small fitness losses from initial WC→GU replacements and large fitness losses from second-site GU→UP replacements, second-site UP→GU replacements are nearly as compensatory as GU→WC replacements at ancestral WC sites in the HIV-1 secondary structure.

This analysis highlights the complexities of epistatic interactions between WC sites in the HIV-1 secondary structure. In particular, although epistatic selection is strong and accounts for nearly half of all constraint on the HIV-1 secondary structure, it primarily targets replacements that completely abolish pairing interactions. In contrast, GU wobbles are typically slightly deleterious and can even compensate for the loss of fitness from initial WC→UP replacements. While the GU wobble as an intermediate is not a novel theme in the evolution of RNA secondary structures, these findings suggest that the GU wobble may play a more central role in compensatory evolution via its ability to “rescue” a RNA secondary structure after an initially deleterious WC→UP replacement. Thus, GU wobbles act not just as intermediates, but also as compensators. Moreover, this study provides the first numerical analysis of the fitness effects of various initial and second-site replacements, including those involving GU wobbles, at WC sites in the RNA secondary structure of HIV-1.

Together, these findings suggest that epistatic selection on the RNA secondary structure of HIV-1 operates under a fitness hierarchy in which 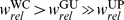, and the ability of a new state to increase in frequency is based on its position in the hierarchy relative to that of the previous state. Because the fitness of a GU wobble is nearly equivalent to that of a WC pair, a GU wobble can be maintained stably at a relatively high frequency in the population, likely until fitness is completely restored by a replacement that re-establishes WC pairing. Thus, GU wobbles compose ridges that connect WC peaks in the epistatic adaptive landscape of the HIV-1 secondary structure, forming relatively flat high-fitness mutational paths to distant peaks. Moreover, while the HIV-1 population will inevitably fall into UP valleys as it traverses the adaptive landscape, it can be rescued from such a valley by a mutation that lifts it to either a WC peak or a GU ridge. Because of the high mutation rate and small genome of HIV-1, such a mutation will arise quickly, preventing the population from becoming trapped in a UP valley and enabling its rapid evolution along the epistatic adaptive landscape.

## Materials and Methods

### Sequence retrieval and analysis

HIV-1 sequences were downloaded from the HIV Sequence Database at http://www.hiv.lanl.gov/ and aligned by HMMER [49] using the HIVAlign [50] tool. The subtype B NL4-3 genome sequence (accession M19921) and corresponding positions of WC pairs in the RNA secondary structure [46] were used as a reference set for all analyses. Protein-coding nonsynonymous sites were removed from analyses to minimize confounding effects of selection on amino acids. However, as expected, findings for a separate analysis of nonsynonymous sites (Figures S1 and S2) are generally consistent with those obtained with their exclusion (Figures 1 and 4). Also, it is important to note that splice sites, which may be under additional selective constraint, were not removed from analyses, although these should not affect overall patterns observed. A site in the reference genome was considered ancestral if it is conserved in all 66 subtype D genomic sequences, and a replacement mutation was inferred when an ancestral site is polymorphic in the subtype B population. WC sites were considered to have undergone a single-site replacement when a polymorphism at one site segregates with the ancestral nucleotide at the interacting site, and a double-site replacement when polymorphisms at both sites segregate together in the population. In cases of double-site replacements, polymorphism frequencies were used to distinguish between initial and second-site replacements. In particular, the polymorphism segregating at a higher frequency (with the ancestral nucleotide at the interacting site) was designated as the initial replacement.

### Normalization of polymorphism frequencies

Selection coefficients were inferred from polymorphism frequencies and the average spontaneous mutation rate of HIV-1. However, mutation rates and effects of selection may vary among different classes of nucleotide replacements. Thus, to enable comparisons of polymorphism frequencies among different classes of replacements, I normalized polymorphism frequencies by multiplying the frequency of each nucleotide replacement by its observed/expected ratio. The expected number of a particular nucleotide replacement (e.g., A→U) was computed by multiplying the number of sites with the ancestral state (e.g., A) by the corresponding nucleotide replacement rate (e.g., A→U), which was estimated from replacements at nWC sites. For example, the A→U replacement rate was computed by dividing the total number of A→U replacements at nWC sites by the total number of nWC sites with replacements of an A (A→U+A→G+A→C). The rates for all replacement types are given in Table S1. As expected, transitions are more common than transversions at nWC sites.

Also shown in Table S1 are replacement rates computed for experimentally derived mutation data from Mansky and Temin (1995) [51]. These rates were not appropriate for the current analysis for two reasons. First, Mansky and Temin did not observe any transversions at three of the four ancestral nucleotides (G, C, and A; see Table S1), which may have been due to their small sample size (42 replacements), and is an unrealistic expectation for the current dataset (7,723 replacements at nWC sites). Second, and more importantly, replacements observed by Mansky and Temin reflect mutation rates, while nWC replacements were likely affected by non-epistatic selection. Thus, I was able to use replacement rates at nWC sites to compare and quantify epistatic and non-epistatic components of selection on the HIV-1 secondary structure, which were major objectives of the current study.

Additionally, because WC→GU polymorphisms segregate at much higher frequencies than WC→UP polymorphisms, and the probability (and frequency) of a particular second-site replacement is proportional to the probability (and frequency) of the initial replacement polymorphism, I normalized frequencies of second-site replacements by median frequencies of single-site replacements.

### Estimation of the proportion of constraint due to epistasis

Selective constraint against replacements at nWC and WC sites are given in Table 1 as *s_nwc_* = 1.26×10^−2^ and *s_wc_* = 3.43×10^−2^. Because *s_wc_* cannot solely be attributed to epistasis, I estimated the epistatic component of *s_wc_* by 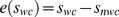. Then, the proportion of *s_wc_* that is due to epistasis can be estimated by 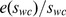, and the proportion of constraint at all HIV-1 sites that is due epistasis can be estimated by 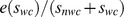, where (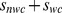) represents total constraint.

### Estimation of fitness changes

Assuming that the optimal genotype has a fitness of 1, the relative fitness (*w_rel_*) of each replacement class can be estimated by 1−*s*, where *s* is the estimated selection coefficient of the respective replacement class that was derived from normalized polymorphism frequencies (see above). Thus, *w_rel_* is the relative median fitness of a particular replacement class based on normalized nucleotide polymorphism frequencies, rather than an experimentally derived fitness value based on viral replication capacity. This estimation enables the comparison of median, but not absolute, fitness effects between replacement classes. The change in relative fitness due to conversion from state 1 to state 2, 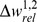, was computed as the difference between the relative fitnesses of the two states (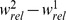), divided by 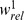. For example, if state 1 is an initial WC→GU replacement and state 2 is a second-site GU→WC replacement, the change in relative fitness due to the second-site replacement is given by
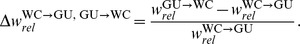



### Statistical analyses

All statistical analyses were performed in the R software environment [52]. An exact binomial test was used to compare mutation saturation levels at WC and non-WC sites by setting *x* to the number of observed polymorphisms at WC sites (1,105), *n* to the total number of possible polymorphisms at WC sites (1,686), and *p* to the proportion of non-WC sites that are saturated by mutation (0.898). Mann-Whitney *U* tests were used to compare all pairs of frequency distributions.

## Supporting Information

Figure S1 Intra-population frequencies of nonsynonymous nWC and single-site WC replacement polymorphisms in the HIV-1 genome. Frequencies are normalized to enable comparisons among classes (see Materials and Methods for details) and plotted on a log_10_-scale. Asterisks indicate *p*<0.05 (*), *p*<0.01 (**), and *p*<0.001 (***).
(PDF)

Figure S2 Intra-population frequencies of nonsynonymous second-site WC replacement polymorphisms in the HIV-1 genome. Frequencies are normalized to enable comparisons among classes (see Materials and Methods for details) and plotted on a log_10_-scale. Asterisks indicate *p*<0.05 (*), *p*<0.01 (**), and *p*<0.001 (***).
(PDF)

Table S1 Nucleotide replacement rates computed from replacements at nWC sites and from experimental mutation data of Mansky and Temin (1995).
(PDF)
