# Supplementary figures and images for: Strong Epistatic Selection on the RNA Secondary Structure of HIV
Source: PLoS Pathog. 2014 Sep 11;10(9):e1004363. doi: 10.1371/journal.ppat.1004363 (PMC4161434; doi:10.1371/journal.ppat.1004363)

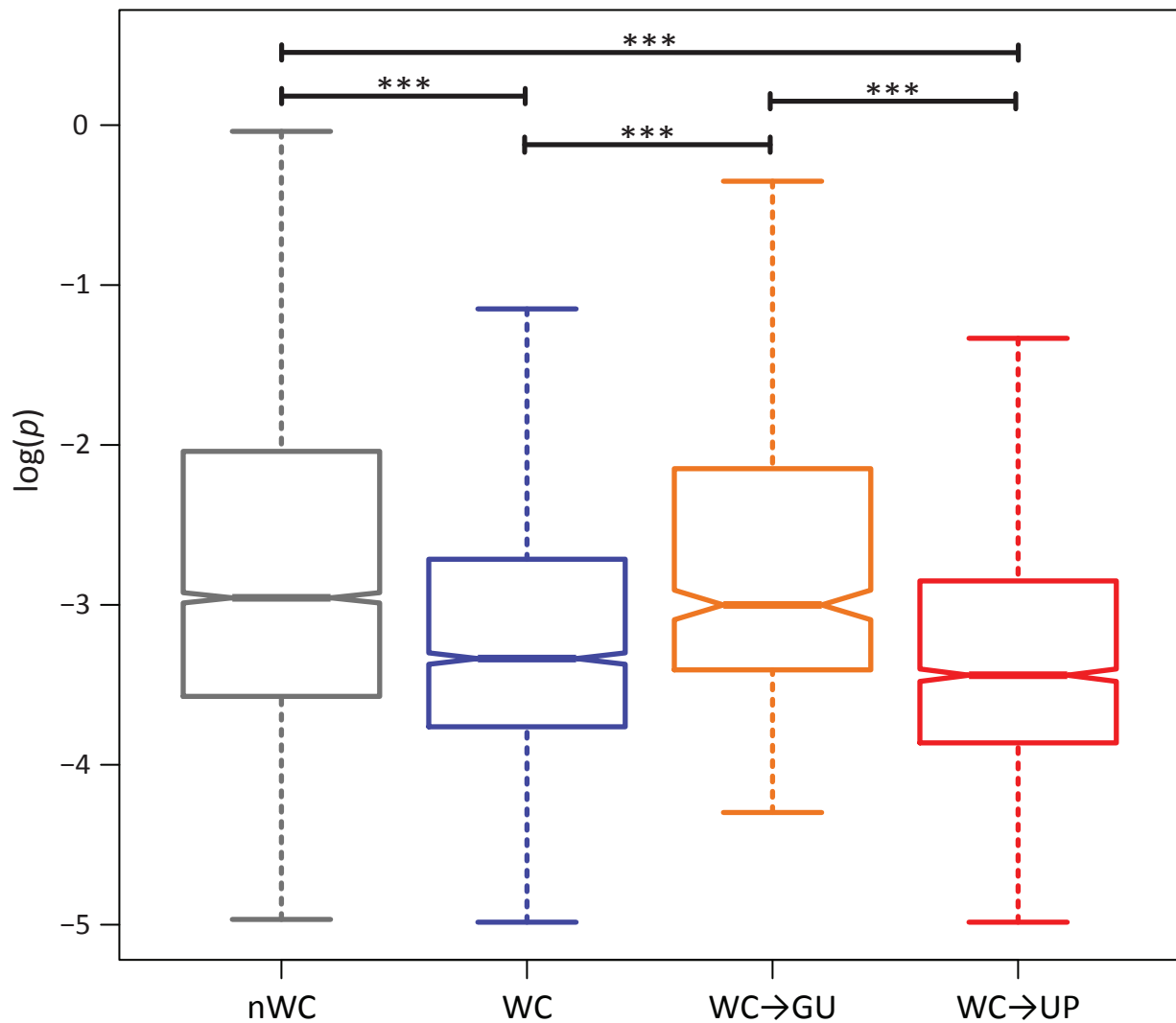

Supplement: Figure S1 — Intra-population frequencies of nonsynonymous nWC and single-site WC replacement polymorphisms in the HIV-1 genome. Frequencies are normalized to enable comparisons among classes (see Materials and Methods for details) and plotted on a log10-scale. Asterisks indicate p<0.05 (*), p<0.01 (**), and p<0.001 (***). (PDF) [file ppat.1004363.s001.pdf]

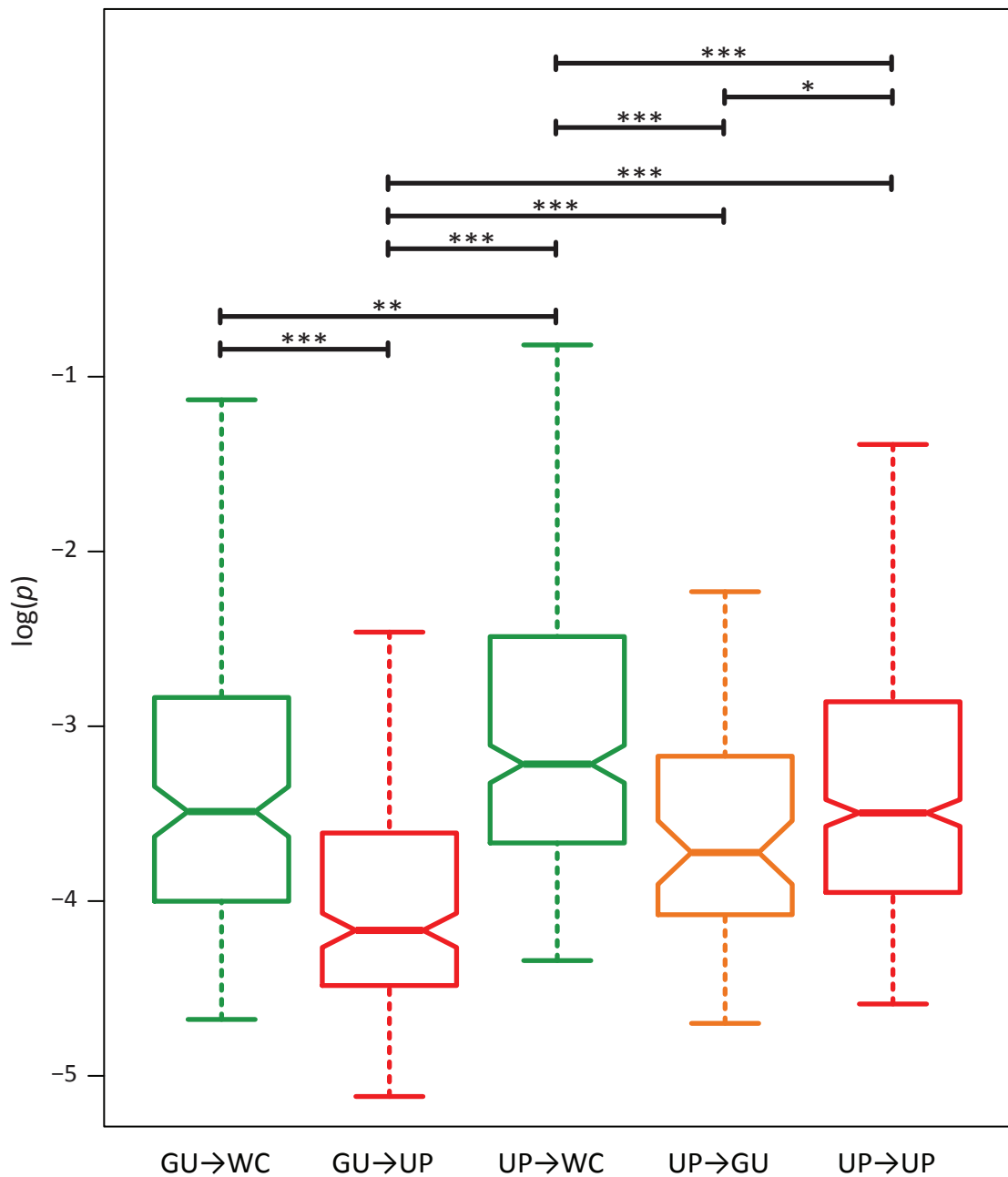

Supplement: Figure S2 — Intra-population frequencies of nonsynonymous second-site WC replacement polymorphisms in the HIV-1 genome. Frequencies are normalized to enable comparisons among classes (see Materials and Methods for details) and plotted on a log10-scale. Asterisks indicate p<0.05 (*), p<0.01 (**), and p<0.001 (***). (PDF) [file ppat.1004363.s002.pdf]
