# Supplementary material for: Strong Epistatic Selection on the RNA Secondary Structure of HIV
Source: PLoS Pathog. 2014 Sep 11;10(9):e1004363. doi: 10.1371/journal.ppat.1004363 (PMC4161434; doi:10.1371/journal.ppat.1004363)
Supplement: Table S1 — Nucleotide replacement rates computed from replacements at nWC sites and from experimental mutation data of Mansky and Temin (1995). (PDF) [file ppat.1004363.s003.pdf]

Table S1. Nucleotide replacement rates computed from observed replacements at nWC sites and from experimental mutation data of Mansky and Temin (1995).

	nWC	Mansky and Temin (1995)
**G→A**	0.39	1
**G→U**	0.30	0
**G→C**	0.32	0
**A→G**	0.36	1
**A→U**	0.32	0
**A→C**	0.33	0
**U→C**	0.37	0.50
**U→G**	0.31	0.25
**U→A**	0.32	0.25
**C→U**	0.37	1
**C→G**	0.30	0
**C→A**	0.33	0
